# The Effects of Emotional States and World Assumptions on Moral Disengagement in Adolescents from Combat Zones

**DOI:** 10.11621/pir.2025.0206

**Published:** 2025-06-01

**Authors:** Alexandra G. Dolgikh, Olga V. Almazova, Sergei V. Molchanov, Ludmila A. Shaigerova

**Affiliations:** a Federal Scientific Center for Psychological and Interdisciplinary Research, Moscow, Russia; b Lomonosov Moscow State University, Russia

**Keywords:** adolescents, negative emotional states, world assumptions, self-worth, benevolence of the world, moral disengagement, mechanisms of moral disengagement

## Abstract

**Background:**

Extreme events, including warfare, lead to transformations in moral norms and the heightened intensity of moral disengagement mechanisms, which may be prolonged or become irreversible. Research to identify the factors that reduce the frequency of moral disengagement mechanisms among adolescents can lead to understanding how to prevent and decrease the intensity of destructive behavior.

**Objective:**

To identify the emotional states and fundamental belief systems that correlate with the intensity of moral disengagement in adolescents from combat zones, compared to peers in a control group.

**Design:**

The study employed the Depression, Anxiety, and Stress Scales (DASS-21) to assess the severity of negative emotional states; the revised World Assumption Scale (Russian adaptation) to measure the intensity of "ve fundamental beliefs; and the Propensity to Morally Disengage Scale (Russian adaptation) to evaluate the extent of moral disengagement. The sample consisted of 196 adolescents aged 13 to 16. 98 participants were residents of a combat zone (Belgorod Region) and 98 participants from various regions of the Russian Federation with no exposure to combat danger.

**Results:**

The study revealed a significantcant deterioration across all assessed indicators of emotional state, as well as a reduction in fundamental beliefs about the benevolence of the world, the trustworthiness of others, and selfworth among adolescents in the primary group compared to the control group. Adolescents from the Belgorod Region exhibited significantly higher levels of 5 (from 8) moral disengagement mechanisms. Predictors of the intensity of moral disengagement were identified.

**Conclusion:**

Living in a combat zone contributes to the intensification of negative emotional states, the breakdown of fundamental belief systems, and a prevalence of moral disengagement mechanisms in adolescents. Depression levels (directly) and beliefs in the benevolence and controllability of the world (inversely) predicted moral disengagement.

## Introduction

Self-determination is a fundamental developmental process during adolescence (Karabanova, 2020), which culminates in the formation of a worldview—a stable “picture of the world encompassing an individual’s perceptions of the natural world, society, their place within it, and the nature of their relationships with others” (Molchanov et al., 2019). A key component of this worldview is one’s system of moral norms and values, which is shaped not only by the microenvironment and the adolescent’s individual habitual circumstances, but also by the distinctive features of the era and society. As L.S. Vygotskiy (1991) notes, “moral concepts and ideas change across social contexts; what was considered harmful in one place and time may be regarded as a virtue in another” (p. 250).

Contemporary society is marked by increasing a diaphorization of human behavior, or moral blindness, which diminishes the importance of moral norms and blurs the lines between “good” and “bad”, as well as “right” and “wrong”, in social relationships (Bauman & Donskis, 2013). The low level of informational security in a modern adolescent’s socialization process influences moral norms and increases the risk of deviant behavior (Karabanova, 2020). The growing importance of social networks in adolescence is accompanied by aggressive communication (Sobkin & Fedotova, 2021). Most adolescents experience different types of cyber aggression from their peers (Soldatova et al., 2020). These shifts are further accelerated by heightened societal transitivity, characterized by significant, rapid, and largely unpredictable changes (Martsinkovskaia & Preobrazhenskaia, 2020; Odintsova et al., 2022). The rising frequency of global and local extreme events, such as armed conflicts and terrorist acts, can lead to the swift and profound reconsideration of entrenched moral norms at both individual and group levels (Aquino et al., 2007), regardless of age.

Adolescence, as a period of self-determination, entails the developmental task of internalizing a system of norms and values that provide guidance in navigating moral dilemmas—a universal aspect of human experience. As Vygotsky, following Piaget, suggests, moral attitudes in adolescence reflect a “mix of elementary social reactions and egocentric impulses” and disrespect for “any moral rules or obligations”, focusing on the idea that moral development, like the development of logic, is a socially driven process (Vygotsky, 1929, p. 468).

Unfavorable social conditions during childhood and adolescence, such as traumatic events, life-threatening situations, and severe restrictions on opportunities, can significantly impact one’s emotional well-being, affect one’s faith in the world, and hinder the ability to build relationships with others. The resulting effects on individuals and groups are often long-lasting and potentially irreversible. For example, adolescents who experienced social violence demonstrate elevated and consistent levels of cognitive distortions for not less than four years afferwards (Esposito, 2020). Regular exposure to extreme experiences—such as the aftermath of armed conflict, acts of cruelty, or systematic dehumanization—can diminish moral sensitivity and foster pronounced moral disengagement in the younger generation.

### Mechanisms of Moral Disengagement

Moral disengagement is the “set of socio-cognitive processes that enable individuals to reinterpret and reframe their behavior to make it appear less harmful”, minimize their responsibility for the consequences of their actions, and avoid the recognition of victims’ suffering (Bandura, 1999). Albert Bandura identified eight core mechanisms of moral disengagement, organized into four loci:

1. Behavior locus: Includes mechanisms such as moral justification, euphemistic labelling, and advantageous comparison.
2. Agency locus: Encompasses displacement of responsibility and diffusion of responsibility.
3. Outcome locus: Involves disregarding or distorting the consequences of actions.
4. Victim locus: Includes attribution of blame and dehumanization (Bandura, 2018).

These loci and their respective mechanisms allow individuals to dissociate self-censure from morally unacceptable behavior through various strategies: justifying harmful actions as benign, focusing on those involved to minimize personal responsibility by diffusing accountability, diminishing or distorting the consequences of their actions, and dehumanizing or engaging in victim blaming (Bandura, 2018). Prosocial motivation in adolescence can be changed due to situational factors (Nartova-Bochaver, 1992). The low resistance of moral norms can lead to immoral behavior (Narvaez & Rest, 1995).

Adolescence is a developmental period during which individuals are particularly prone to developing mechanisms of moral disengagement, compared to other age groups (Bandura, 2015; Luo & Bussey, 2023). This is evident in the growing body of research on aggressive behaviors in adolescent settings, such as bullying and cyber-bullying (Bussey, 2023). A meta-analysis of 47 studies involving over 43,000 children and adolescents demonstrated a consistent link between the activation of moral disengagement mechanisms and bullying during adolescence (Killer et al., 2019).

Both perpetrators and bystanders frequently employ moral disengagement mechanisms in peer bullying (Thornberg, 2023), in cyberbullying (Zhao & Yu, 2021), during interactions in collaborative online gaming (Ak et al., 2022), and as a collective means of mitigating guilt in situations of widespread moral norm violations (Gini et al., 2020).

### Negative Emotional States in Adolescence

Compared to adults, adolescents experience both positive and negative emotions with greater intensity and exhibit higher levels of emotional tension and instability. While the intensity of positive emotions tends to gradually decline, the intensity of negative emotions remains stable throughout adolescence (Bailen et al., 2019; Reitsema et al., 2022). Experiencing stress can become an important contributor to negative emotions (Krapic et al., 2015). The increasing frequency and intensity of negative emotional states are often reflected in heightened depressive symptoms, pronounced reactions to stressors, and elevated anxiety related to typical life events during this stage of development, such as important exams, making life decisions, separating from parents, and the growing importance of peer relationships (Östberg et al., 2015).

Emotional instability is a regular phenomenon in the transition from childhood to adulthood and arouses difficulties in coping with negative emotions in adolescents. A lack of timely diagnosis and intervention can lead to serious physical and mental health issues, adversely affecting all areas of life, including relationships with peers and family members. Furthermore, it can contribute to the emergence of deviant behaviors (Pascoe et al., 2020; Santee & Starr, 2022; Schleider et al., 2021).

### Fundamental Assumptions

Assumptive World Theory proposed the idea that individuals possess a stable set of assumptions about themselves, others, and the world around them, based on their life experience (Janoff-Bulman, 1989, 1992). The theory identifies eight core beliefs grouped into three primary categories: the benevolence of the world, the meaningfulness of events, and self-worth (Janoff-Bulman, 1989). These beliefs are initially shaped during early childhood, based on interactions with family or other caregivers, and generally don’t sustain significant change due to minor, quotidian adversities. However, traumatic events can profoundly disrupt even firmly established assumptions (Janoff-Bulman, 1992).

Traditionally, the system of basic assumptions and their role have been studied in relation to trauma, particularly in children and youth (Ferrajão & Elklit, 2023). For instance, a study involving university students found a persistent relationship between fundamental beliefs and depressive symptoms in those who had experienced interpersonal trauma. Specifically, depressive symptoms were three times more pronounced in individuals with a perceived lack of safety in the world (Schleider et al., 2021). The quantity of population-based studies of fundamental beliefs is increasing. Some of them are focused on adolescents and examine their relationships with other psychological phenomena and constructs. For example, a study of Russian adolescents analyzed the theoretical correlation between fundamental beliefs and components of C. Ryff’s (2014) model of psychological well-being. The study demonstrated the conceptual similarity of constructs such as self-acceptance and self-worth, control over external circumstances and life controllability, positive relationships with others, and the benevolence of the world (Molchanov et al., 2019).

The goal of the present study was to investigate the characteristics of emotional states and fundamental beliefs, and their interaction with moral disengagement mechanisms in adolescents from combat zones, compared to their peers in a control group.

The study explored the following research questions:

1. Are there differences in negative emotional states between adolescents from combat zones and those from the control group?
2. Are there differences in the fundamental beliefs between adolescents from combat zones and those from the control group?
3. Are there differences in the level of moral disengagement between adolescents from combat zones and those from the control group?
4. Can negative emotional states and fundamental beliefs be considered predictors of prioritization of moral disengagement mechanisms in adolescence?

## Methods

### Participants

The participants were 196 adolescents aged 13 to 16 years (M = 15.1; SD = 1.03). The primary group consisted of 98 teenagers (29 boys and 69 girls) who permanently reside in the Belgorod region, a combat zone. The control group included 98 adolescents (29 boys and 69 girls) who, at the time of the study, were applicants to the Advanced Educational Scientific Center – Kolmogorov’s Boarding School of Moscow State University (AESC MSU) and resided in various regions of the Russian Federation.

Adolescents from the Belgorod region were surveyed four days after their arrival at a summer camp in the Moscow region. They had been offered a short-term recreational program for academic achievement. The Belgorod region has been subjected to systematic shelling, resulting in destruction of property and loss of life.

The control group travelled to Moscow from different regions of Russia to apply to study at AESC MSU—a highly competitive educational institution that offers free tuition for gifted students—and to participate in MSU’s summer school. They were surveyed on the third day of their arrival, prior to the start of entrance examinations.

### Procedure

#### Questionnaires

A pen-and-paper questionnaire survey was conducted in 2024.

The *Depression Anxiety Stress Scales (DASS-21)* (Lovibond & Lovibond, 1995) was used in order to assess the negative emotional states experienced by respondents over the past week. This version of the questionnaire consists of 21 items, scored on a 4-point Likert scale, ranging from: 1 (“strongly disagree”) to 4 (“strongly agree”). Analysis shows that this scale can be used with an adolescent sample with some limitations (Jovanovic et al., 2021).

Fundamental beliefs were assessed using the revised *World Assumption Scale (WAS)* (Janoff-Bulman, 1989) in a Russian adaptation (Padun & Kotelnikova, 2008). Unlike the original version of the methodology, which consisted of eight sub-scales, the revised Russian version highlights *five dimensions* of basic individual assumptions: benevolence of the world, justice, self-worth, luck, and controllability. A total of 37 items were scored on a 6-point scale, from1 (“strongly disagrees”) to 6 (“strongly agrees”). The World Assumption Scale has been successfully used in research with adolescents (Karabanova et al., 2018).

In order to measure the intensity of eight moral disengagement mechanisms, as described by Bandura (2015), the Russian adaptation of the *Propensity to Morally Disengage Scale (MD-24)* was used (Ledova et al., 2016; Moore et al., 2012). A total of 24 items were scored on a 7-point scale, from1 (“strongly disagrees”) to 7 (“strongly agrees”). The Scale was successfully used in research with adolescents (Molchanov & Almazova, 2020).

#### Statistical Analysis

All analyses were performed using Jamovi version 2.3.28. The Shapiro-Wilk test indicated that the variables followed a normal distribution; therefore, we used parametric tests. To examine Research Questions 1–3, Independent Samples t-test was conducted to evaluate the differences in assessments of emotional states, basic assumptions, and the frequency of use of disengagement mechanisms in adolescents from the two groups. To answer Research Question 4, linear regression was used. Assessments of various aspects of emotional states and assumptions were used as predictors, and frequency of use of each locus of moral disengagement mechanisms were use das dependent variables in each linear regression.

An a priori power analysis was conducted using G*Power version 3.1.9.4. (Faul et al., 2007) to determine the total sample size required to detect a significant effect if one exists. The results indicated that the required sample size to achieve 80% power for detecting an effect between small and medium (Cohen’s *d* = .35), at a significance criterion of α = .05, was *N* = 200 for the Independent Samples t-test (100 people in each group). There quired sample size to achieve 80% power for detecting a small effect (η2 = .02), at a significance criterion of α = .05, was *N* = 109 for linear regression. Thus, the sample should consist of 200 teenagers. A post hoc power analysis was conducted, as the obtained sample size (*N* = 196) was fewer than 200. However, the obtained sample size was sufficient for linear regression purposes. Therefore, the obtained sample size was adequate to answer the research questions.

## Results

At the primary stage, a comparative analysis of the features of negative emotional states, basic assumptions, and the intensity of moral disengagement mechanisms was conducted for the main and control groups of teenagers. Subsequently, the relationship between indicators of negative emotional states and basic assumptions with the intensity of moral disengagement mechanisms was examined.

### Emotional States Indicators and Intensity of Basic Assumptions

*Table 1* shows the means and standard deviations for the scores on the three DASS-21 scales and the five WAS scales for both respondent groups, as well as the results of these score comparisons (independent samples *t-test*).

**Table 1:** Means and Standard Deviations for Scores on the DASS-21 and WAS Scales in Adolescents from Two Groups, Comparison of Scores

Group / DASS-21 and WAS criteria	Belgorod region		AESC MSU		Differences		
	*M*	*SD*	*M*	*SD*	*t* (*df*=194)	*p*	Cohen’s *d*
Depression	9.1	9.37	5.8	7.10	2.91**	.004	.418
Anxiety	10.8	10.06	5.1	6.93	4.72**	<.001	.677
Stress	14.4	10.06	10.2	7.60	3.40**	<.001	.488
Benevolence of World (BW)	3.7	.94	4.2	.87	-3.05**	.003	.438
Justice (J)	4.0	.65	3.7	.94	2.54*	.012	.365
Self-Worth (SW)	4.0	.73	4.5	.97	-4.17**	<.001	.599
Luck (L)	3.9	.70	4.3	.73	-3.46**	<.001	.497
Controllability (C)	3.9	.54	4.3	.54	-4.99**	<.001	.717

Note. * p<.05; ** p<.01

Since the sample size was fewer than 200 respondents (a priori power analyses showed that for Independent Samples t-test with the size of the effect .35, the sample size should be *N*= 200), post hoc power analysis was conducted. For the smallest parameter effect with a significant difference (the Justice scale, Cohen’s *d*=.37) results indicated that Power (1-β error probability) = .82, which is more than 80%, which suggests that the results obtained are reliable. Thus, the sample size of *N* = 196 was enough to detect a significant effect.

The results indicate that all indicators of negative emotional states–depression, anxiety, and stress–are significantly higher among adolescents from the Belgorod region than in the control group. Analysis of the basic belief scores revealed that adolescents from the Belgorod region scored significantly lower on four out of the five WAS scales (benevolence of world, self-worth, luck, and controllability) and significantly higher on the justice scale, compared to the control group.

### Moral Disengagement Mechanisms in the Two Groups

Table 2 presents the mean values and standard deviations of the expression of moral disengagement mechanisms in adolescents from the experimental and control groups, as well as the results of comparisons of these measures using the *t*-test for independent samples.

**Table 2:** Mean Values and Standard Deviations of Scores on the MD-24 Questionnaire Scales in Adolescents from the Two Groups, and Comparison of Scores

Group / Mechanisms of moral disengagement	Belgorod region		AESC MSU		Differences		
	*M*	*SD*	*M*	*SD*	*t* (*df=t* 194)	*p*	Cohen’s *d*
Moral Justification	3.3	1.17	3.5	1.28	–1.13	.261	.166
Euphemistic Labelling	2.9	1.13	2.6	1.21	1.97*	.050	.290
Advantageous Comparison	2.4	0.95	1.8	0.90	3.73**	<.001	.548
Displacement of Responsibility	2.9	1.17	2.2	1.03	4.34**	<.001	.637
Diffusion of Responsibility	3.0	1.16	2.6	1.26	2.35*	.020	.346
Disregard of Consequences or Distortion	2.7	1.09	2.1	1.07	4.24**	<.001	.623
Dehumanization	4.0	1.29	3.7	1.45	1.63	.105	.240
Attribution of Blame	2.7	1.05	3.5	1.28	4.64**	<.001	.682

Note. * p<.05; ** p<.01

Since the sample size was fewer than 200 (a priori power analyses showed that for Independent Samples t-test with the size of the effect .35 sample size should be *N* = 200), post hoc power analysis was conducted. For the smallest parameter effect with a significant difference (the diffusion of responsibility, Cohen’s *d*=.35) the results indicated that Power (1-β error probability) = .77, which is less than 80%, and therefore does not allow us to say that the results are reliable. For other mechanisms with significant differences, the effect size is larger than .37, for which the reliability of the results was established above. Thus, the sample size of *N* = 196 was enough to detect a significant effect for all results except those regarding the mechanism of diffusion of responsibility.

We found that the assessment for most of the moral disengagement mechanisms — advantageous comparison, displacement of responsibility, diffusion of responsibility, disregard or distortion of consequences, and attribution of blame — are significantly higher in adolescents from the Belgorod region compared to those in the control group. No significant differences were found between the two groups for the mechanisms of moral justification, euphemistic labelling, and dehumanization.

### Predictors of the Intensity of Moral Disengagement Use

To identify the predictors of moral disengagement according to Bandura’s theory (Bandura, 2018), the eight mechanisms were grouped into four loci: 1) behavior locus, 2) agency locus, 3) outcome locus, and 4) victim locus, with the values for each locus calculated as the arithmetic mean of the ratings for the mechanisms within it.

Linear regression analysis was conducted to identify predictors of the intensity of the moral disengagement loci using the scores from the DASS-21 (Depression, Anxiety, Stress Scales) and WAS (World Assumption Scale) (Table 3). Pearson correlation analysis revealed that in both groups—the combat zone group and the control group — the significant relationships between the three constructs under consideration were highly similar, allowing us to construct general regression models.

Since the sample size does not allow for separate regression analysis for the main and control groups, the analysis was performed for the entire sample.

**Table 3:** Mean and Standard Score Deviations on the DASS-21 and WAS Questionnaires for Adolescents in the Two Groups: Comparison of Scores

Moral disengagement locus/ DASS-21 and WAS	Behavior locus (*R*^2^ = 0.433; *p* < 0.001)		Agency locus (*R*^2^ = 0.407; *p* < 0.001)		Outcome locus (*R*^2^ = 0.394; *p* < 0.001)		Victim locus (*R*^2^ = 0.420; *p* < 0.001)	
	Beta	*p*	Beta	*p*	Beta	*p*	Beta	*p*
Depression	.245*	.039	.120	.316	.277*	.022	.082	.488
Anxiety	–.046	.734	–.034	.802	–.006	.966	–.153	.258
Stress	–.034	.795	.015	.909	–.120	.368	.186	.159
Benevolence of World (BW)	–.280**	.005	–.203*	.041	–.265**	.008	–.307**	.002
Justice (J)	–.104	.190	.070	.381	.051	.529	.027	.735
Self-Worth (SW)	.059	.541	.066	.501	.115	.242	.075	.441
Luck (L)	.026	.759	–.041	.635	.037	.676	–.004	.961
Controllability (C)	–.046	.592	–.277**	.002	–.195*	.025	–.170*	.08

Note. * p<.05; ** p<.01

The following results were obtained:

- The intensity of the use of mechanisms within the behavior locus is predicted directly by the level of depression (43.3% of variance) and inversely by the benevolence of the world.
- The intensity of the use of mechanisms within the agency locus is predicted inversely by the benevolence of the world and the controllability of one’s life (40.7% of variance).
- The intensity of the use of mechanisms within the outcome locus is predicted directly by the level of depression (39.4% of variance) and inversely by the benevolence of the world and controllability.
- The intensity of the use of mechanisms within the victim locus is predicted inversely by the benevolence of the world and controllability of one’s life (42.0% of variance).

## Discussion

First and foremost, the results of the study allowed for the identification of the characteristics of negative emotional states, fundamental beliefs, and the use of mechanisms of moral disengagement in adolescents from combat zones, and their comparison with the results of the control group. The findings indicate that both the main and the control group are more prone to stress than to depression and anxiety, which may point to a general trend of stress dominance during adolescence compared to other negative emotional states (Krapic et al., 2015; Östberg et al., 2015). However, the two groups display differences in the levels of all studied negative emotions (depression, anxiety, stress), where they are significantly higher in adolescents from the Belgorod region compared to the control group, which suggests that the current situation poses a major threat to their mental health. At the same time, such heightened emotional intensity may serve as an adaptive function in adolescence, acting as a social signal to those around the teenagers, indicating the need for support (Bailen, Green, & Thompson, 2019). It should also be noted that a study of a student sample showed that respondents who left the combat zone more often exhibited self-doubt, mistrust of the world, anxiety, irritability, and a feeling of helplessness (Tkachenko & Mishina, 2024).

Analysis of differences in the basic beliefs showed that four out of the five beliefs examined are significantly less pronounced in adolescents from the main group compared to the control group. This suggests that belief in the benevolence of the world, belief in self-worth, belief in luck, and belief in the controllability of life have been significantly diminished in adolescents from the Belgorod region due to their life experience. The only belief that is significantly higher in the main sample compared to the control group is the belief in justice, which dominates the overall system of their basic beliefs. This result is particularly interesting in light of the fact that belief in justice occupies the lowest position in the hierarchy of other basic beliefs in the control sample, which corresponds to findings from other studies involving adolescents (Molchanov & Almazova, 2020), whereas the dominant belief in the control group is a positive self-image (self-worth). The dominance of the belief in the justice of the world among adolescents from combat zones requires further interpretation, but the data used in this analysis does not allow for the construction of a mathematically substantiated explanatory model. The fact that self-worth is diminished in this group of adolescents, especially when contrasted with the control group, warrants close attention. Self-worth is a component of the self-concept, which includes not only a person’s self-representations, but also their subjective perception of external factors that influence their personality (Burns, 1982). The process of self-knowledge, which determines the development of the self-concept, always occurs within a specific social environment (Bozhovich, 2001). Changes in the habitual environment, the sudden emergence of direct life threats, and confrontation with the limitations imposed by the proximity to a conflict zone inevitably affect the process of self-knowledge in adolescence (Zinchenko, 2013). Negative transformations in the environment may lead the adolescent to experience a sense of limitation in their ability to influence what is happening, feelings of a lack of control over their life, helplessness, and dependence on circumstances (Zinchenko et al., 2021), which, in turn, lead to a reduction in self-worth.

Analysis of moral disengagement mechanisms showed similar tendencies for both groups: the mechanisms of dehumanization of the victim and moral justification of one’s own behavior are most pronounced, while the mechanism of advantageous comparison is the least pronounced. Similarly, Bandura (2002) notes that adolescence is characterized by the predominance of the mechanisms of moral disengagement and victim dehumanization, alongside the mechanism of the diffusion of responsibility, which in our sample is expressed at an average level (in both the main and control groups). The difference between the two groups of adolescents is evident in the significantly greater expression of most disengagement mechanisms (five out of eight) in adolescents from the Belgorod region compared to the control group: advantageous comparison, displacement of responsibility, diffusion of responsibility, disregard or distortion of consequences, and attribution of blame. This established pattern of cognitive schemes may indicate that living in a combat zone creates the need, first, to accept and explain the contradiction of the changed worldview, prompting adolescents to rely on mechanisms such as advantageous comparison, disregard or distortion of consequences, and attribution of blame. Second, the adolescents’ awareness of their helplessness in the face of the situation and their inability to influence events that are incompatible with the idea of a just world contributes to a more pronounced tendency to shift and diffuse responsibility (displacement and diffusion of responsibility), which in this context may serve as a protective mechanism aimed at maintaining a positive self-image, the value of which has been diminished, both in comparison to the control group and relative to the other beliefs in their basic belief system.

Numerous studies in various socio cultural contexts have found that the use of moral disengagement mechanisms can predict deviant behavior in adolescents (Gini et al., 2020; Killer et al., 2019; Thornberg, 2023; Zhao & Yu, 2021), which is a signal for that timely measures should be taken to prevent mass criminalization or deterioration of the mental health of an entire generational cohort.

Analysis of the relationship between the four loci of moral disengagement and negative emotional states and basic beliefs shows that three indicators (two of the basic beliefs and one of the three negative emotional states) act as predictors of the expression of moral disengagement. Differences between the two adolescent groups in the significance of these predictors were not analyzed due to the insufficient sample size, but it can be assumed that their role in the context of shaping the moral consciousness of the younger generation is important in any context, both in relatively stable conditions and especially in contexts associated with increased risks to health and life, given the significantly more pronounced negative emotional states and basic beliefs in adolescents from conflict zones compared to the control group.

The most significant predictor in this context was the basic belief in the benevolence of the world, which acts as a reverse predictor for the expression of all four loci of moral disengagement. Therefore, it can be suggested that belief in the benevolence of the world and of others contributes to the formation of a more highly developed moral consciousness in adolescents, while its loss leads to moral disengagement. The second most important predictor, with the most connections to moral disengagement, is the belief in control over one’s life (controllability), which is the only one not related to one of the four loci—destructive behavior. For the other three loci, controllability acts as a reverse predictor, suggesting that adolescents’ belief in their ability to control events also plays an important role in the development of morals and ethics.

The third predictor of moral disengagement is the level of depression, which directly predicts the expression of moral disengagement mechanisms related to two loci—behavior locus and outcome locus. Thus, it was found that a higher level of depression in adolescents can contribute to a reduction in moral principles in terms of behavioral manifestations, including verbal behavior, as well as the assessment of harm caused to others. Specifically, there is evidence that during adolescence, depression symptoms are linked to a greater expression of moral disengagement mechanisms in bullying situations (Wang et al., 2017).

The obtained results have high practical significance and can be applied in psychological practice aimed at preventing the risks of deviant behavior during adolescence and the erosion of moral and ethical principles. The content of an individual’s moral norms and principles is socially conditioned. Moral behavior in children and adolescents, “just like any other behavior … is developed under the influence of systematic environmental factors” (Vygotsky, 1991). It is well known that living in a conflict zone is often accompanied by increased aggression levels, a rise in deviant behavior, and heightened use of moral disengagement mechanisms (Bandura, 2015). The findings of this study can contribute to stabilizing the emotional state of adolescents living in combat zones (particularly in reducing depressive symptoms), fostering their belief in the possibility of controlling the world around them, including through the recognition of its benevolence, and strengthening their moral self-awareness.

## Conclusion

The findings indicated a significant deterioration in emotional state, lower levels of trust in the world and in others, and in self-belief, as well as a greater expression of self-justification mechanisms in adolescents from the Belgorod region. All the emotional state indicators (depression, anxiety, stress) were significantly higher in those residing in the combat zone compared to their peers from the control group. The indicators of the majority of the considered basic beliefs—four out of five (benevolence of the world, positive self-image, belief in luck, and belief in control over life)—were lower in adolescents from the conflict zone than in the control group, indicating a pronounced tendency towards the destruction of beliefs in the benevolence of the world, the ability to control events, and the value of the self. Additionally, eight out of the five self-justification mechanisms were expressed more strongly. Depression levels (directly), belief in the benevolence of the world, and belief in control over one’s life (inversely) were identified as predictors of the intensity of moral disengagement mechanisms.

## Limitations

The limitations of the study may be determined by the characteristics of the sample. Both the main and control groups included high-achieving high school students who were motivated to succeed, which may indirectly reflect a social micro-situation conducive to the moral and cognitive development of adolescents and support from family, friends, and significant others. Several studies have shown the key mediating role of parents and peer friends in the development of moral identity and the use of moral disengagement mechanisms (Li, 2020; Wang et al., 2020). The system of relationships between the adolescents and their immediate social environment, which constructs the social micro system of development, may serve as a factor mitigating the negative, destructive impact of violence and aggression prevailing in the social macrosystem on emotional well-being and moral development. Thus, the results may not reflect the characteristics of adolescents in less favorable conditions, although significant differences in emotional state, basic beliefs, and moral disengagement were observed even in a favorable sample from the combat zone.

Additionally, the limited sample size of adolescents from the conflict zone, due to the challenges of recruiting respondents, did not allow for a comparison of the identified predictors of moral disengagement between the main and control groups, nor for an analysis of gender differences. The small sample size also prevented us from conducting separate regression analyses for each subsample. The predominance of girls in the sample precluded an analysis of gender differences.

Another limitation is the lack of available information regarding the level of involvement of the teenagers from the Belgorod region in the consequences of the armed conflict they are currently facing. It can be assumed that the level of engagement of adolescents and their families in the ongoing events may differ, which, in turn, could influence the differences in emotional states, basic beliefs, and moral disengagement mechanisms.
